# From the Exposure Model to the Generative Brain: Art, Creativity, and the Case for Cultural Vitality as Health Infrastructure

**DOI:** 10.3390/brainsci16101104

**Published:** 2026-10-14

**Authors:** Claudio Lucchiari, Maria Elide Vanutelli

**Affiliations:** 1 Department of Philosophy “Piero Martinetti”, Università degli Studi di Milano, 20122 Milan, Italy; 2 Department of Psychology, University of Milan-Bicocca, 20126 Milan, Italy; maria.vanutelli@unimib.it

**Keywords:** neuroaesthetics, art, well-being, social prescription, neuro-driven cultural change, cultural vitality

## Abstract

**Highlights:**

**What are the main findings?**

- We advance the hypothesis that aesthetic reception and creative production are two directions of a single, neurally instantiated generative regime, scaling from a transient individual brain state to dyadic (C^2^) and group-level creativity.
- If the regime is not confined to the brain but is instead a coupled brain–environment system, the health-relevant unit of analysis is the environment as well as the individual, which makes cultural vitality a candidate determinant of health rather than an established one.

**What are the implications of the main findings?**

- Social-prescribing models that treat cultural engagement as a dose delivered to an individual patient are mismatched to an epidemiological signature that resembles a cultivated, distributed capacity; their weak evidence base is read here as a symptom of that mismatch rather than of poor implementation.
- Three explicit criteria and seven falsifiable predictions are specified so that the infrastructural status of cultural vitality can be tested, and if unsupported, rejected, before policy commitments harden.

**Abstract:**

A quarter-century of neuroaesthetics and cultural epidemiology has established that engagement with art is broadly and durably health-bearing, yet the finding sits awkwardly within the paradigm through which it is usually read: a tacit exposure model, in which art is beneficial input, the person is its recipient, and benefit is modelled on the dose received. Translated into practice as social prescribing, now expanding on an evidence base that the field’s own systematic reviews describe as weak, this model inherits the grammar of the receptor and the individual patient, and leaves an anomaly it cannot explain. The epidemiological signature of cultural engagement is that of a cultivated capacity distributed across a coupled system rather than of an administered agent; it survives adjustment for socioeconomic position, health status, health behaviours, and social connectedness, though not the residual confounding we treat as its most serious rival explanation. We advance the hypothesis that aesthetic reception and creative production are two directions of a single generative regime of brain function: neurally instantiated but neither neurally nor individually bounded, scaling from a transient individual state to dyadic and group-level creativity and to the enactive coupling of organism and environment. Cultural vitality thereby becomes a candidate infrastructural determinant of health, adjudicated by three stated criteria and tested through seven falsifiable predictions. Whether the reclassification holds is an empirical question, and one that neuroscience is morally compelled to help resolve before policy commitments harden.

## 1. An Anomaly a Quarter-Century in the Making

The turn of the millennium marked art’s formal entry into neuroscience. With the coining of neuroaesthetics [1] and the later articulation of aesthetic experience as the coordinated engagement of sensory–motor, emotion–valuation, and meaning–knowledge systems, the aesthetic triad [2], the artwork ceased to be merely an object of humanistic interpretation and became a stimulus complex through which cognition itself could be probed. Twenty-five years on, two largely separate bodies of evidence have accumulated, and it is their juxtaposition, rather than either alone, that generates the problem this paper addresses.

The first body of evidence is mechanistic and, almost without exception, acute. Peak aesthetic pleasure in music tracks phasic dopamine release in the striatal reward circuit [3]; states of intense aesthetic absorption recruit the default mode network in a manner atypical for externally directed tasks, as though the artwork were being processed as self-relevant rather than as an external object [4]; single museum or laboratory exposures measurably lower physiological and self-reported markers of stress and anxiety [4,5,6,7,8,9,10]. These are robust findings, but they share a grammar: one participant, one session, one stimulus, one outcome, measured minutes to hours after exposure.

The second body of evidence is epidemiological and chronic. Population cohorts link habitual cultural engagement to lower incidence of depression, slower cognitive decline, and, in the English Longitudinal Study of Ageing, reduced all-cause mortality across a decade of follow-up [11]; the WHO scoping synthesis catalogues benefits across an extraordinary range of conditions and life stages [12]. Whether these associations are an artefact of who chooses to engage is the first question to put to them, and adjustment answers it only in part. The mortality association survives adjustment for socioeconomic position, health status, health behaviours including physical activity, and social connectedness, with each factor individually accounting for only a small fraction of the effect [11]. For the parallel association with depression, socioeconomic status explains roughly half of the effect, but neither moderates it nor accounts for it entirely, with the independent association confirmed across logistic regression, propensity-score matching, and fixed-effects analyses [13]. Here, the grammar is entirely different: whole populations, lifetimes, heterogeneous exposures, distal outcomes.

Residual confounding is the most serious rival explanation for the anomaly we are describing, better stated at its strongest than managed. A broad-spectrum association with health and no isolable active ingredient is, after all, also the classic signature of confounding. The plausible candidates are not exhausted by the covariates ordinarily adjusted for the following: a healthy-user effect, whereby people already disposed towards self-care engage more; cognitive reserve, and the quality rather than the duration of education; mobility, which conditions engagement and health alike; neighbourhood resources; personality; and reverse causation from subclinical decline, which can reduce engagement years before it registers as disease. Adjustment for a measured set of covariates does not rule these out, and the propensity-score and fixed-effects analyses cited above narrow the space without closing it. What this literature currently lacks, and what we would urge upon it, is routine reporting of sensitivity to unmeasured confounding. An E-value, quantifying how strong an unmeasured confounder would have to be, on the risk-ratio scale, to explain away an observed association, would render the robustness of these estimates comparable across studies and would discipline claims of the kind we are advancing here.

Access to healthcare is one such candidate, and we single it out because it is the one most often raised against arts-and-health findings and the one least often measured. To our knowledge, no cohort study has entered it as a baseline covariate. Four observations bear on it indirectly, and we offer them as such rather than as a refutation. The mortality association is observed within universal-coverage systems, as in the Norwegian HUNT cohort [14]; universal coverage, however, equalises entitlement rather than effective access, which continues to vary with distance, health literacy, and trust. At the ecological level, cultural participation has been found to predict public health efficiency, an outcome defined relative to healthcare resource inputs, in a specification absorbing stable city-level differences in health infrastructure [15]; that outcome is not itself a health outcome, and the estimates await independent replication. Community-level social constructs can dissociate from healthcare access rather than travelling with it, as among rural older adults who report higher neighbourhood cohesion yet poorer access and worse health status [16]; that study does not concern cultural engagement, and the parallel it supplies is structural rather than evidential. Engagement has also been reported to precede and shape later patterns of healthcare utilisation [17], which bears on the direction of the relation without establishing it. Taken together, these observations make access to care an unlikely sole explanation of the association; they do not make it an improbable contributor, and only a design in which it is measured at baseline, as P5 specifies, can decide the matter.

The anomaly appears when these two grammars are read against a single, tacit model of how the brain relates to art, the model that in fact underwrites much of the field and, more consequentially, its translation into policy. On that model, which we call the exposure model and characterise in Section The Opponent Position, and What It Is Not, art is a source of beneficial input received by an individual, and its benefit should therefore behave like that of an administered agent: identifiable in its active ingredient, graded in its dose, specific in its effect. The epidemiological signal behaves nothing like this. Its effects are strikingly broad in spectrum: mood, cognition, stress physiology, immune parameters, loneliness, longevity, for an exposure with no isolable active ingredient: painting, choral singing, theatre-going and dance point in the same salutary direction despite sharing almost no proximate mechanism. Its dependence on engagement is graded, but in the manner of a cultivated capacity rather than a titrated substance, cumulative across the life course, sensitive to early and habitual participation, embedded in identity and social context. Cultural engagement, in short, carries the epidemiological fingerprint of things like physical activity, education, or social connection, capacities a person develops and maintains, and not that of a drug a clinician delivers.

Between these two studies lies a conspicuous gap, which we will call the missing link: we possess acute mechanisms and distal outcomes, but almost nothing that connects a single dopaminergic or default-mode event to a decade of altered mortality risk. The exposure model offers no natural bridge across this gap, because within it the acute event simply is the benefit, merely repeated. We will argue that the gap is a symptom rather than an accident, that the paradigm misdescribes what art does to the brain, and that a better description dissolves the anomaly and specifies the missing link at once. Doing so requires us to bring in a phenomenon the exposure model treats as unrelated, and which the remainder of this paper places at its centre: not only the art we receive, but the art we make. We call the resulting position a neuro-driven cultural change, meaning a revision in how health systems classify cultural life that is driven by evidence from the brain sciences rather than by cultural advocacy; Section 4 states the direction of that inference precisely, since the phrase invites the opposite reading.

### The Opponent Position, and What It Is Not

The brain presupposed by the paradigm we criticise is not the one of predictive processing. In that framework, perception is framed as constructive and anticipatory, grounded in a generative model that the organism uses to explain its sensory stream; accordingly, a purely reactive brain is logically untenable. A substantial literature has already applied this framework to aesthetics, from early prediction-error accounts of visual art [18] to recent active-inference treatments of the aesthetic episode as an epistemic arc of curiosity, epistemic action, and resolution [19]. We take these accounts to be broadly correct about what they set out to explain, and we do not contest them.

Our target is narrower: not a theory of brain function, but the tacit model that governs the translation of arts-and-health findings into practice. On that model, which we will call the exposure model, art is a stimulus, the person is its recipient, and benefit is a function of how much is received. It is this, and not any Bayesian commitment, which underwrites the search for an active ingredient, the expectation of a graded dose, and the referral of an individual patient to a quantity of cultural activity. The exposure model is not an account of brain function, and we attribute it to no one. It is an operating assumption embedded in practice: absent from the literature as an explicit position, yet legible in intervention protocols, in dosing decisions, and in the design of evaluations. Therein lies its resilience, since an assumption that is never stated is never examined. Its persistence in policy, rather than its standing in cognitive science, is what makes it worth naming.

What, then, does a generative regime add to an account in which the brain is already conceived as generative? The distinction we intend is between a generative model and a generative regime. Predictive processing attributes a generative model to the brain at all times and in all states. We propose that a particular, transient configuration of that machinery carries the properties relevant to health, and that access to this configuration is unevenly distributed and modifiable. Three additions follow, each separable from the others and each independently defeasible.

First, symmetry. Predictive-processing accounts of art are, with few exceptions, accounts of the beholder: the epistemic arc is traced by a perceiver confronting an artefact that someone else has made. Active inference has the resources to treat making as well, since action is itself inference, but the aesthetics literature has not developed the productive direction. We hypothesise that reception and production are one regime run in opposite directions, and recent evidence on a left inferior-parietal technical-cognition system recruited both when people make and when they observe or are taught making [20,21] gives this claim a substrate that can be tested rather than assumed.

Second, cultivability. The epistemic arc explains an episode. It does not, by itself, explain why access to the regime should be trainable, why it should accumulate across a life course, or why its stability in later life should be mediated by crystallised semantic resources [22]. We define the generative regime as a state with a modifiable access threshold, and it is this property, rather than the episode, which connects to an epidemiological signature of cultivated capacity.

Third, scale. Active inference admits multi-agent formulations in which interacting agents converge on shared generative models [23]. We take these as support rather than competition: they supply a formal vocabulary in which the dyadic and collective extensions proposed in Section 3 can be stated, and in which the inter-brain predictions can be given a mechanistic reading.

The generative regime, on this reading, is not a rival to predictive processing. Rather, it specifies which states, and at what scales and temporal persistence, carry the properties that matter for health. This way, the proposal is formulated in explicitly falsifiable terms.

## 2. Two Directions of One Regime

We propose that the relevant object is neither the artwork nor the reaction to it, but a regime of brain function that art characteristically engages. This regime is internally generated rather than stimulus-clamped, oriented toward the construction of meaning rather than the extraction of information, anticipatory rather than consummatory in its reward dynamics, and regulative of the internal milieu as much as representational of the external one. We call this the generative regime, and its signature is a reversed direction of fitness: rather than the brain modelling itself on the world, the world is recruited as material for a model the brain is building.

Figure 1 sets out the structure of the proposal as a whole: the two directions, the signature that defines the regime, the scales across which it is hypothesised to extend, and the environmental term that governs access to it. Crucially, this regime has a describable functional signature, and the signature is one of timing rather than of a static pattern of activation. Entry into the regime requires a phase in which internally generated, associative processing provisionally prevails over premature evaluative control, releasing the long-range associations that routine processing suppresses. What must follow, however, is a reciprocal exchange, in which executive systems re-engage to select, test, and shape what the associative phase has produced. Creative cognition accordingly arises not from default-network activity alone but from the controlled coupling of the default network with executive systems, a finding supported not only by correlational connectivity analyses but by causal evidence from intracranial stimulation, neurofeedback, and transcranial modulation [24,25]. This two-phase structure matters because it specifies what the regime is not. Internally generated activity that is never met by such re-engagement does not constitute the regime: it is fantasy, or, where its contents are negatively valenced and recursively constrained, rumination, a state marked by sustained and unrelieved default-network dominance and associated with poorer rather than better mental health [26]. The distinguishing signature is therefore neither default-network recruitment nor executive engagement taken alone, but the time-varying alternation and coupling of the two; its absence is indicated by internally generated activity that remains uncoupled across the task window. This is also what distinguishes the proposed regime from internally oriented processing in general, and it makes the distinction empirically decidable rather than stipulative. What is distinctive about art is not that it engages this regime; prospection and simulation do too, but that it is the one cultural domain in which the regime runs in both directions, since we are in it when we make, and when we are moved.

### 2.1. The Productive Direction

The idea that generativity is a general human capacity rather than the endowment of exceptional minds is the settled lesson of half a century of creativity research: Runco’s emphasis on creative potential and everyday (little-c) creativity, and Simonton’s combinatorial account of generativity as recombination under constraint, both locate creativity in ordinary cognition. We have argued that this capacity is best understood not as a stable trait but as a transient configuration of the cognitive system, a creative-on state entered through a phase in which default-network processing provisionally prevails over the evaluative control exercised by inferior-frontal and anterior-temporal systems, releasing the long-range semantic associations that routine, creative-off processing suppresses, and sustained thereafter by the reciprocal coupling of associative and executive systems described above [27]. The view that creativity is a capacity rather than a trait is independently supported by lifespan evidence: creative performance on divergent-thinking tasks remains stable into older adulthood even as the associative processes that feed it decline, with the stability mediated by the accumulated semantic repertoire that a life of cultural engagement builds, that is, the crystallised intelligence [22]. Two features of the creative-on account matter here. First, the state is evoked and trainable, not merely possessed: it can be triggered by task and context, and access to it can be cultivated. Second, productive direction does not terminate in ideation but presses toward realisation, a need-for-enactment that couples the generative regime to motor programmes and yields an external object. The made artwork is the generative regime taking material form.

### 2.2. The Receptive Direction

The same regime, we argue, underlies aesthetic experience, not as a separate faculty but as the receptive orientation of a single capacity. Intense aesthetic experience recruits the default network in a manner atypical for externally directed perception, as though the work was processed as self-relevant rather than as an external object [4]. We believe that to be moved by an artwork is to regenerate its meaning rather than to receive it. Reception, on this account, is the generative regime run in the receptive direction of fit: the beholder is a maker who supplies the model that the artwork is recruited to complete. This has a methodological corollary. Choosing to study cognition through complex, self-defining artistic stimuli rather than through simple repeatable ones already embodies a claim about what the brain is doing with art, and the choice should be defended as such.

### 2.3. The Collective Dimension

The generative regime is not confined to a single brain, and this is what converts cultural infrastructure from slogan into a tractable object. At the dyadic level, two engaged brains do not produce a sum of two individual creativities but a non-linear combination, a genuine two-person unit of generativity that we have termed C^2^, detectable in principle as inter-brain synchronisation and productive dissonance and naturally described within an extended-mind framework [28]. We state this as a proposal rather than as a result. Inter-brain synchrony can be generated by shared sensory input and by common task structure in the absence of any genuine coupling, a confound the hyperscanning literature treats as central, and separating C^2^ from those alternatives requires designs that decorrelate shared input from joint generation. Until such designs are run, the construct remains to be validated. The notion that inter-brain coupling is a real and measurable phenomenon rather than an analogy is now established independently, from brain-to-brain coupling in communication [29] to classroom EEG synchrony tracking shared engagement [30]. At the group level, generativity can be defined and measured as a property of the collective itself: we have shown experimentally that class-level creative capacity can be raised by training in primary-school children, with gains registered in the group’s aggregate performance rather than in isolated individuals [31], consistent with evidence that collective intelligence is an emergent property of group interaction, not a sum of member abilities, and one that can itself be cultivated through training [32]. The regime, in short, exists at the scale of the state, the dyad, and the group; and at every scale it is exercised and cultivated rather than administered. It is precisely this fact, that generative capacity is trainable at the collective level and embedded in the social and material environment, that the concluding sections translate into a claim about infrastructure.

### 2.4. From Repeated Access to Distal Outcome

If the predictions set out below are to follow from the hypothesis rather than merely sit well with it, the pathway from an acute state to a distal outcome must be stated. We propose one route, and we explicitly state what it does not cover.

Each episode of regime access exercises the coupling described above and draws on the semantic repertoire that the associative phase recruits, enlarging and reorganising it (hypothetical). Accumulated across a life course, that repertoire is the crystallised resource that mediates the stability of generative performance in later life [established]. Crystallised resources of this kind are constituents of cognitive reserve [construct established; its dependence on regime access hypothetical], and cognitive reserve predicts slower functional decline and lower all-cause mortality (established). Figure 2 sets out the route and the evidential status of each link.

The predictions follow from this route rather than accompanying it. P1 tests its first premise, that what matters is generative demand rather than quantity of exposure. P2 tests whether the acute signature forecasts and mediates the distal trajectory, which is what any pathway claim requires. P3 and P4 ask whether the same route operates when the regime is distributed across brains. P5 to P7 ask whether provision governs the rate at which the route is travelled at all.

This route does not explain the cardiovascular associations reported in Section 1, and we do not claim that it does. A regulatory pathway is conceivable: entry into the regime requires the provisional release of premature evaluative control, and the state it opens is incompatible with the recursively constrained processing characteristic of rumination, so repeated access might reduce the time spent in that mode and with it chronic stress load. Every link in that chain is at present untested, including the first. We record it as a direction for work rather than as part of the hypothesis, and none of the predictions advanced here depends on it.

## 3. From a Regime of the Brain to a Regime of the Brain–Environment System

Ascending the three scales of the generative regime does not merely enlarge it; it changes what kind of thing the regime is, and this is not a claim we need to establish from our own data. Dynamical accounts of creative behaviour have argued that the degrees of freedom participating in a behavioural pattern are situated beyond the boundaries of the skull and flesh of an agent, since neurobiological agents are not closed systems but maintain themselves through the couplings they establish with their environment [33]. On this view, a creative shift is not the activation of a dedicated module but a reorganisation of a coupled system: established patterns lose their hold, uncertainty rises, and the field of available actions widens. The same formalism has been shown to operate at the interpersonal level, where partners function as degrees of freedom of a single system and the novel patterns that emerge belong to none of them individually. Independently, and from an entirely different literature, reviews of collective creativity converge on the conclusion that collective outcomes are governed by variation and integration across a collective’s cognitive, social and organisational architecture rather than by the properties of its constituent units [34]. Two fields with no shared vocabulary thus arrive at the same displacement of the explanatory locus.

Our own findings sit within this convergence. At the level of the single state, the creative-on configuration can still be described as a property of one brain [27]. A dyadic C^2^ unit, by construction, cannot: it is a property of the coupling between two brains, which is why it must be measured between them rather than within either [28]. And class-level creativity cannot be located in any single participant at all; in our primary-school training study the capacity that improved was defined only over the group, its gains registered in an aggregate performance that no individual score reproduces [31].

These results invite an obvious objection: an aggregate score may simply reflect the sum of what the members brought to the task, in which case nothing has emerged at all. The question of whether group-level capacities are reducible to individual ones has been posed most directly in research on collective intelligence, where a general factor of group performance was found to emerge from patterns of interaction and to be statistically independent of the individual abilities of the members [35]. That result concerns performance broadly rather than creativity specifically, and it has been read as settling the point at issue here, namely that what is defined over a group need not be derivable from those within it. That reading now requires qualification. Subsequent work has recovered the factor only weakly or has found it largely explained by the individual abilities of the members, so irreducibility is better treated as an open question, and as one that prediction P4 is designed to test rather than to presuppose.

The generative regime is therefore present at every neural level, but it is not confined to the brain. As it scales, the focus shifts to the coupled embodied brain–environment system, which enactive and extended approaches consider the proper unit of mind [36,37,38]. Participatory sense-making claims precisely that coupling itself can carry meaning, and that meaning can be enacted between agents rather than only within one [39]. That is the theoretical counterpart of what C^2^ and class-level creativity show empirically.

## 4. From Prescription to Infrastructure

Policy is moving faster than the evidence that would justify it, and the direction it has taken follows from the model we have been contesting. The main way the evidence from Section 1 has been translated into practice is social prescribing: referring individuals to cultural activities as part of their care. Its aims are valuable, and some of its outcomes are real. However, this approach does not fully capture the process described in the previous sections. It treats cultural activity as something that can be prescribed to an individual, like a treatment given in a specific dose. But cultural engagement depends on the interaction between the whole organism and its environment. The relevant conditions are therefore not simply clinical or individual, since they are also environmental and collective, shaping a population’s capacity to create meaning. The evidence base that social prescribing has accumulated is, by the field’s own assessment, weak and inconsistent. A systematic review of eight trials found no consistent effect of social-prescribing link workers on quality of life or mental health outcomes [40], though that review concerns link-worker models generally, not arts-based referral specifically. Meanwhile, a recent narrative review concluded that the model risks individualising structural disadvantage by shifting the burden of managing multimorbidity onto those least equipped to respond [41]. Recent evidence suggests this is not simply a matter of poor targeting: social-prescribing referrals in a representative English cohort disproportionately reach those with the greatest socioeconomic need, loneliness, and multimorbidity, although some geographic inconsistency in provision remains [42].

We read this fragility as a symptom of the mismatch identified above rather than as a defect to be corrected with better trials. That reading is a hypothesis, and it is not the only one available. Heterogeneity in intervention content, implementation fidelity, referral pathways, local availability of activities, follow-up duration, and outcome selection would each explain inconsistent effects without any appeal to a mismatch of paradigms. Consistency requires us to state what evidence would favour that alternative over ours, and three patterns would. First, trials that hold content, dose, and follow-up constant and thereby recover consistent individual-level effects where earlier trials did not: in that case the problem lay in design. Second, a reliable, graded relation between the quantity of prescribed activity and individual outcomes, holding independently of the properties of the surrounding environment. Third, individual-level effects that persist where environmental provision is poor. Our own reading predicts the opposite pattern: effect sizes for identical referrals will vary systematically with the cultural provision available around the person referred, while environment-level measures will explain variance that individual dose does not. If that is right, it carries an uncomfortable corollary for how public money is currently spent: evaluations that measure only what is prescribed, and not the provision surrounding the person to whom it is prescribed, are designed in a way that cannot recover the effect they were commissioned to find. The corollary does not require anyone to accept our account of the brain. It requires only that the environment be measured alongside the referral.

Environmental exposures can and do show graded relations with health; air quality and green space are measured in precisely this way. We argue that the dose which matters here is not administered to an individual on a given occasion, and that a design measuring only what is prescribed will not recover it.

By cultural vitality, we denote a property of an environment, separate from the characteristics of the individuals within it: specifically, the rate at which such an environment affords and sustains opportunities for generative brain–environment coupling in both receptive and productive directions (Section 2). Since this definition rests on environmental affordances rather than observed outcomes, the construct requires a measurement model that does not presuppose what it seeks to explain. We detail it below. This is deliberately narrower than three adjacent constructs with which it is easily conflated. Cultural engagement denotes an individual’s behavioural exposure—how often a person attends or participates—and is a measurable correlate of cultural vitality, not a synonym for it: an environment can sustain high engagement while affording little generative renewal, and vice versa. Social capital denotes a relational resource among people—trust, reciprocity, network ties—and is conceptually orthogonal: cultural vitality can be high in a setting of weak social ties and low in a setting of strong ones. Social infrastructure, in Klinenberg’s sense [43], denotes physical and civic spaces that enable social contact in general; cultural vitality is a narrower claim about the density of generative sense-making opportunities specifically, which such spaces may or may not house.

Defining cultural vitality in terms of generative coupling raises an obvious objection: if the construct is measured by the coupling it is intended to explain, it cannot be used to test claims about that coupling. The remedy lies in separating the construct from its indicators. Accordingly, we specify cultural vitality as a latent property of a locality, estimated from supply-side indicators drawn from administrative and geospatial records. The index captures cultural provision, not participation or engagement. One further consequence is that provision is also not generative coupling. We hypothesise that it is a valid proxy for the rate of coupling an environment affords, and that proxy relation is not assumed but tested, since it is the convergent-validity claim stated as P7. Should it fail, the index measures provision and should be called by that name. These indicators make no reference to neural activity, behaviour, or health, and can be registered independently of any outcomes.

We argue that at least four dimensions, each contributing indicators that existing cultural statistics and mapping infrastructures either already collect or could gather at minimal cost.

Provision: Per capita density of cultural participation occasions within an area, weighted toward participatory activities (making, rehearsing, performing) rather than passive attendance, along with diversity of formats.

Reach: The proportion of occasions that are free or below a defined cost threshold; their accessibility by public transport within a specified travel time; scheduling outside standard working hours; and physical and linguistic accessibility.

Threshold: The proportion of provision requiring no membership, prior booking, training, or demonstrated competence, since occasions available only to the already initiated afford little to outsiders.

Renewal: The rate at which an area’s cultural programme changes, measured as new or substantially altered occasions per year, thereby operationalising the requirement to renew rather than merely maintain.

However, a fifth distributional dimension may be added: the gradient linking provision to area deprivation, since an index blind to the location of provision would classify as vital an area whose resources are concentrated away from those least served.

Two aspects of this specification are crucial to the argument. The first is that it makes the construct defeasible. If the full index predicts population health no better than a bare count of venues does, then the additional dimensions carry no information and the construct, as we have defined it, adds nothing to what cultural-provision mapping already offers; we would take that as a refutation rather than as an invitation to reweight. The second is that it converts the circularity into an empirical question. Because the index is estimated independently of any measure of coupling, the relation between the two can be tested: whether areas scoring higher on the index actually host more of the generative coupling described in Section 2 is a matter of convergent validity, to be established with the ecological methods set out below, and it may fail. Only if it stands does the index earn the name we have given it. Weights across the four dimensions should be fixed and registered in advance of any outcome analysis, for the obvious reason that an index tuned on the outcome it predicts has predicted nothing.

The alternative is to treat the cultural environment as what it is: an infrastructure. It depends on the vitality of the cultural environment within which embodied minds enact their worlds, just as population health depends on the built environment, air quality, green space, and the density of social connection, determinants that operate below the level of any individual decision and are secured structurally or not at all. In this context, the focus of the policy is not the patient but the environment; not the referral but the service; not the therapeutic interaction but the enduring conditions that facilitate generative connection throughout a lifespan and across a community. Cultural access ceases to be a matter of enrichment or leisure and becomes a determinant of health in the technical sense, with the corollary that unequal distribution is a structural health inequality of the same kind as unequal access to clean air or safe streets.

We take a determinant to be infrastructural, rather than an activity, exposure, or intervention, when it satisfies three conditions. First, it operates at population scale: it shapes exposure for whoever is within its reach, not for a selected recipient. Second, it is structurally secured: its presence or absence is fixed by policy, funding, or design, not by an individual’s choice on a given occasion—this is what separates infrastructure from a service that a person elects to use. Third, it is non-rival within capacity and ambient, in the manner of other congestible public goods such as roads or parks, whose finite capacity does not disqualify them as infrastructure. This is not made of discrete units administered one at a time, but of a standing condition that raises or lowers the probability of generative engagement for everyone exposed to it. Social prescribing, on this criterion, is not itself infrastructural—it is a referral mechanism that presupposes an infrastructure already exists to refer people into. Whether cultural vitality meets these three conditions in practice is precisely what Table 1 and Table 2 are designed to test.

What would it mean to act on this? In the first instance, we need to treat the cultural environment as an object of structural investment and measurement rather than of discretionary funding. Encouragingly, the raw materials for such a shift already exist. Within our own programme (the ASBA project), a standardised contemplative protocol delivered in two ordinary Milan museums, an art gallery and a natural-history museum, produced large, immediate reductions in state anxiety across both settings, using existing gallery space and no specialised equipment [6,44]. Interestingly, portable neurophysiological recording in the same settings not only tracked those effects but predicted individual responsiveness from a resting EEG marker [5]. These are small, acute, single-session studies, and we present them as exactly that. Their value here is twofold: they show that the generative coupling we have described can be measured where it actually occurs rather than only in the laboratory, and they make concrete what an environment-level, low-threshold, population-facing provision might look like in practice.

However, a clear assessment of the evidence is more useful than advocacy, and the evidence currently shows that we do not yet have what the infrastructural claim ultimately requires: medium- and long-term data linking sustained cultural engagement to brain and organismic health across the life course. Our own studies measure acute change and explicitly stop there. The population associations that reach across decades are epidemiological and cannot, on their own, identify mechanisms. The link in-between is missing. We see this not as a weakness, but as a concrete implication of the argument: it suggests a research programme, with testable predictions in Table 2, and two key commitments. The first is methodological. The neuroscience marshalled here remains overwhelmingly acute, laboratory-based, and single-brain; a museum is not a scanner, and a theatre is not a lab, and the field will not close the missing link until it can measure generative coupling where it lives. This is a call for sustained investment in ecological methods, portable and mobile neurophysiology of the kind our museum studies piloted, dyadic and group hyperscanning of the sort C^2^ was designed to enable, and longitudinal cohorts embedded in real cultural settings. This choice is not meant to make cultural life more laboratory-like, but to make science more life-like. Ecological validity is the precondition of studying this phenomenon at all.

The second commitment concerns what science is for, and it is the one we most want to stress. Cultural engagement is easily dismissed as elite enthusiasm, a discretionary taste, pleasant for those inclined to it and beside the point for public health. It is precisely here that neuroscience discharges a responsibility that is social, not merely epistemic. By showing that engagement with a vital cultural environment affects the core systems involved in stress regulation, reward, plasticity, and sense-making, this research identifies cultural vitality as a determinant of long-term health. Its unequal distribution is therefore a question of justice, rather than simply a matter of personal preference. That distributional question is, at present, largely unanswered: access to cultural vitality is already known to be patterned. Longitudinal data from the English Longitudinal Study of Ageing show that older adults in lower wealth groups access substantially fewer cultural activities than their more affluent peers despite comparable or greater levels of depressive symptoms, and that this gap has widened over the past decade [45]. If anything, early evidence suggests such benefits may be at least as strong, and possibly stronger, for those facing greater deprivation [46], making the patterning of access a matter of foregone benefit as well as of fairness. A neuro-cultural account of health implies that such gradients are health inequalities and warrant measurement and intervention as such. What the argument settles is only the conditional: if it is unequally distributed, it is a matter of health equity. If science shows how culture supports health but does nothing when cultural vitality is treated as a luxury or unequally distributed, it has fulfilled its scientific role but failed its social responsibility.

These predictions are not exhaustive but are chosen to span the three scales of the generative regime (individual, dyadic/collective, population) and the three temporal grains the missing link requires (acute, medium-term, chronic). The falsification criterion for each prediction is specified in the rightmost column of Table 2; jointly, they are capable of disciplining the paradigm they are designed to test. Three of these predictions are constrained by what existing data can support, and we state the constraints rather than leave them to be discovered. P1 is only partly testable in ELSA and SHARE, which index the frequency of engagement far better than the generative demand of the occasions engaged in; testing it fully requires additional items. P5 depends on longitudinal registers of venue openings, closures, and funding decisions, which exist in usable form in some national contexts and not in others. P7 depends on portable ecological neurophysiology applied to area samples, which is feasible today but has not yet been attempted at that scale. Where the data are not yet adequate, the prediction identifies what would have to be collected.

None of these predictions is decisive on its own. A null result will most often falsify an operationalisation rather than the claim above it, which is why the final column of Table 2 distinguishes the two. The framework as a whole would nevertheless be defeated by a specific conjunction: if no time-resolved signature distinguishes generative engagement from matched non-generative activity, and inter-brain and group-level measures add nothing to individual ones under pseudo-dyad and multilevel control, and an index of cultural vitality neither tracks measured coupling nor predicts health under quasi-experimental identification, then there is no generative regime in the sense we have proposed, and the reclassification argued for here should be abandoned.

This is what a neuro-driven cultural change amounts to, and the direction of the inference must be stated precisely, because the phrase invites the opposite reading. The claim is not that art is valuable because it benefits the brain. That would reduce culture to its neurological effects, which the enactive approach rejects. The relationship works the other way around. Art and creativity are vital because they allow embodied minds to create meaning; because they are vital in this sense, they also support health. The brain sciences and embodied cognition do not confer value upon culture. Instead, they challenge the idea that culture is optional for health and show that cultural vitality is part of the infrastructure that societies must either maintain or allow to decline. The focus here is not really about art; rather, it is on how a health system and a culture are obliged to classify what art is.

Two caveats limit this claim. First, it assumes a value judgement that the argument does not itself establish, i.e., that the conditions for well-being should be fairly distributed, rather than available only to those who happen to have access to them. Science can establish that cultural vitality is such a condition; that we ought therefore to secure it is a commitment we hold openly, not a deduction from data. Second, infrastructure is not a panacea. A vital cultural environment neither substitutes for the material determinants of health nor guarantees well-being on its own: it is a necessary condition of enacting well-being, not a sufficient one, and it stands alongside the other structural determinants, not in their place. Neither caveat softens what is at stake. If the account set out here survives the tests we have specified, a health system that treats cultural life as discretionary is not merely undervaluing it: it is misclassifying a determinant of health, and misclassification at that level has consequences that can be counted.

## 5. Conclusions

A quarter-century of research placed art inside life sciences and then, reasonably enough, asked what it is good for. A large corpus of research suggests that cultural engagement can have broad, cumulative, and lasting effects on health. Those results have been framed within an exposure model, in which art is a beneficial input, and the person is its recipient. The same model underlies social prescribing, where cultural activity is treated as a discrete intervention delivered to individuals in a specific dose. We have argued that this reading leaves a missing link. The epidemiological signature of cultural engagement is not that of an administered agent, but of a cultivated capacity, and a capacity, moreover, that does not sit wholly inside any single brain.

The reframing we have proposed dissolves the anomaly by correcting the unit of analysis. Aesthetic reception and creative production are two directions of one generative regime. It is instantiated in the brain but bounded by neither the brain nor the individual, scaling from the creative-on state through the dyadic non-linearity of C^2^ to the trainable creativity of the group. From an enactive perspective, the relevant subject is not the brain alone, but a living organism that creates meaning through its interaction with an environment rich enough to support it. On the hypothesis advanced here, brain health, organismic health, and the vitality of the cultural environment would, at this scale, be three descriptions of one system rather than three separate objects. That is the claim we put forward for testing, not a result we report. Neuro-driven cultural change means recognising cultural vitality not as a neurological instrument, but as fundamental to health itself.

If that description holds, a practical claim follows from it: what sustains health would be less like a dose to be prescribed than like an environment to be maintained, and cultural vitality would qualify as infrastructure in the technical sense, a determinant whose unequal distribution would constitute a health inequality and whose upkeep would be a public responsibility.

The evidence needed to support this reclassification remains unavailable. Collecting it through ecological and longitudinal research on coupled and collective brains defines the research programme that flows from these arguments. Advancing this case, and accepting the social responsibility it entails, not merely the epistemic one, is work the brain sciences cannot delegate to the humanities alone. We can put the matter still more sharply: the reclassification we propose may prove inappropriate, but what is indefensible is settling the question by default. Allowing provision to contract and referrals to expand while the decisive evidence remains uncollected is itself indefensible.

## Figures and Tables

**Figure 1:**
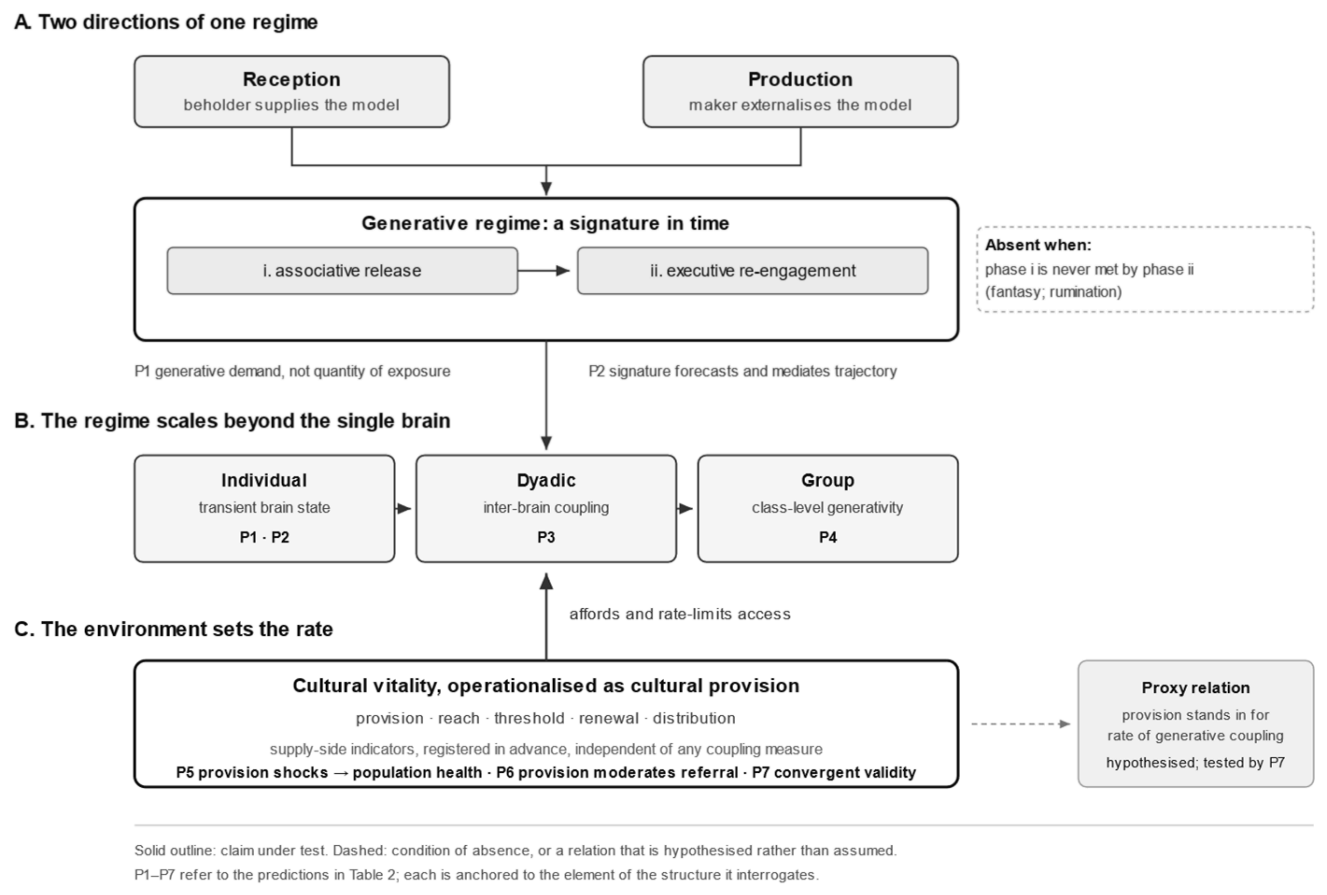
The generative regime: two directions, one signature, three scales, and the environmental term that sets the rate of access. Solid outlines mark claims under test; dashed elements mark the condition under which the regime is absent, and the proxy relation between cultural provision and generative coupling, which is hypothesised rather than assumed. P1–P7 refer to the predictions in Table 2.

**Figure 2:**
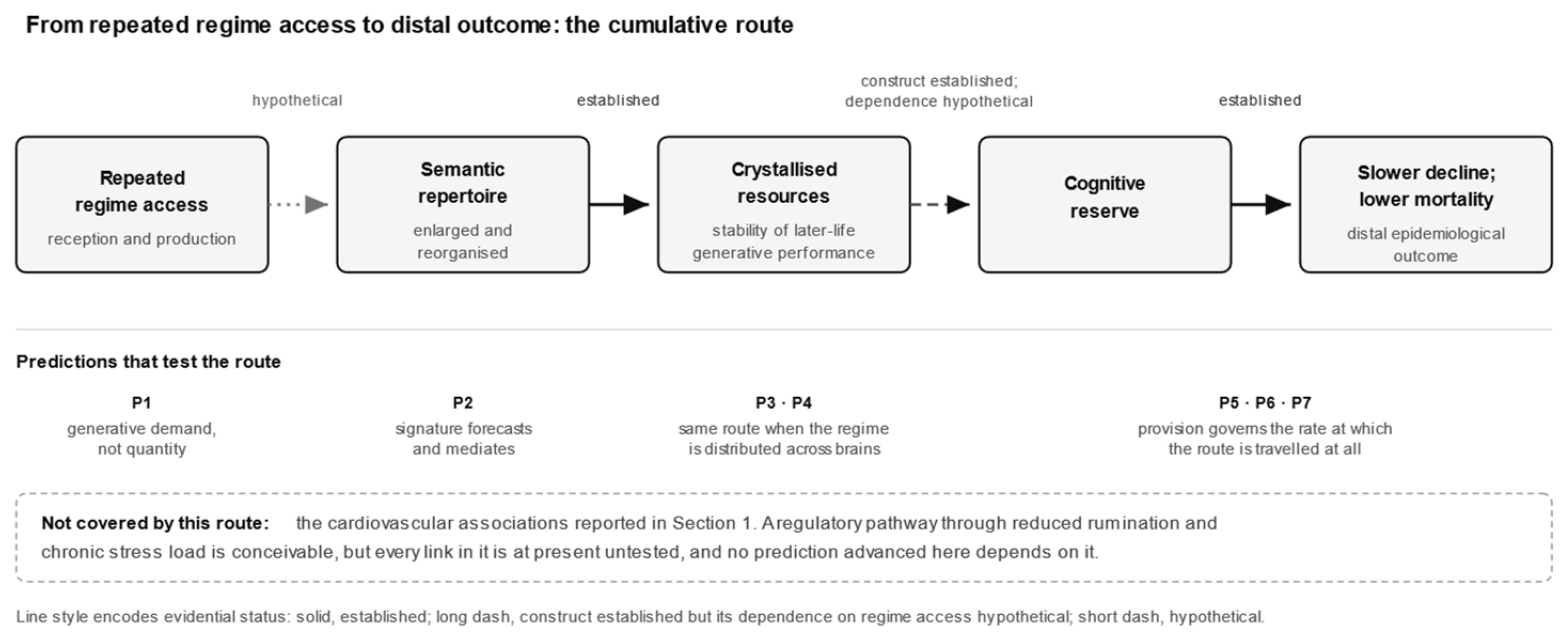
The cumulative route from repeated regime access to distal outcome, with the evidential status of each link encoded by line style: solid, established; long dash, construct established but its dependence on regime access hypothetical; short dash, hypothetical. The route does not cover the cardiovascular associations discussed in Section 1.

**Table 1:** The inferential chain from established findings to the hypothesis under test. Rows are ordered by scale, from the individual state to the population level. For each row, the table states the finding, the strength of the evidence supporting it, the theoretical step that extends it, and the proposition that the framework leaves open for testing. Evidential status is labelled as established, suggestive, or hypothetical, so that steps of unequal evidential weight are not read as equivalent.

Established Finding	Evidential Status	Theoretical Bridge	Hypothesis Under Test
Individual. Creative-on as time-varying DMN–executive coupling; causal evidence from stimulation, neurofeedback, modulation [24,25,26,27]. Aesthetic experience recruits DMN [4].	Established for production. Suggestive for reception: DMN recruitment does not identify the regime (reverse inference).	One regime, two directions; shared technical-cognition substrate as the hinge [20,21]. See Figure 1.	A common time-resolved signature in both directions; acute markers forecast later outcomes (P1, P2).
Dyadic. Inter-brain synchronisation during joint creative activity covaries with joint performance [28].	Suggestive; construct proposed, not validated. Shared input and common task structure are live alternatives.	The dyad as a unit of generativity; licensed by dynamical-systems and extended-mind accounts [36,37,38] and multi-agent active inference [23].	Inter-brain measures add predictive variance beyond individual ones (P3).
Group. Class-level creative performance improves after group training, with gains defined over the group [31,32].	Suggestive; irreducibility not shown. Collective-intelligence evidence [35] is contested.	Outcomes located in variation and integration across a collective’s architecture [34]; step taken by structural analogy.	Group-level physiological change not reducible to individual gains (P4).
Population. Cultural engagement is associated with broad, durable health outcomes [11,14].	Established as association; observational. Residual confounding and reverse causation remain live (Section 1).	Consistent with, but not establishing, a pathway through the regime; route specified in Section 2.4.	The association is carried by generative demand rather than frequency alone (P1).
Structural determinants operate at population level, independently of individual choice.	Established for recognised determinants; membership of cultural vitality hypothetical.	Cultural vitality as candidate member: population scale, structural securing, non-rivalry within capacity.	Provision shocks predict population health under quasi-experimental designs (P5).

**Table 2:** Predictions derived from the generative-regime framework, grouped by the central claim each is designed to test: C1, that reception and production are two directions of one regime; C2, that the regime scales beyond the single brain; C3, that cultural vitality is an infrastructural determinant of health. For each prediction, the table states the design that would test it, the result that would falsify it, and what a null result would and would not take with it.

#	Claim	Prediction	Design	Falsification Criterion	Scope of a Null
P1	C1	At matched frequency, duration, sociality, and energy expenditure, occasions requiring active construction of meaning show stronger associations with health than matched occasions that do not, within the receptive and the productive mode alike.	Cohorts, within-person (fixed effects) on starting and stopping; requires items indexing generative demand.	No gradient by generative demand under matching; or a productive advantage that disappears under matching.	This operationalisation of C1. C1 survives if P2 holds.
P2	C1	The two-phase signature (Section 2) measured during ecological engagement forecasts well-being trajectories, and repeated measurement mediates the association.	Portable EEG/fNIRS in situ; ≥ 3 waves over 6–24 months. One wave tests prediction, not mediation.	No association with later trajectories; or association unchanged when the signature enters as a time-varying mediator.	A null on mediation leaves prediction intact; a null on both removes the mechanism of C1.
P3	C2	Inter-brain synchrony adds predictive variance for group-level outcomes beyond individual measures, in real but not pseudo-dyads.	Dual/multi-brain recording with pseudo-dyad surrogate analysis.	No added variance; or no difference between real and pseudo-dyads.	C^2^ as a unit of generativity. C2 may survive via P4.
P4	C2	Group training produces physiological change whose class-level variance component survives conditioning on individual baseline and gain.	Pre-registered school trial, multilevel models. Salivary cortisol as minimum; hair cortisol, vagally mediated HRV, inflammatory markers as finer alternatives.	Class-level variance component indistinguishable from zero after conditioning.	Irreducibility at group level. A null on one marker removes that operationalisation only.
P5	C3	Changes in cultural provision are followed by changes in population health.	Difference-in-differences on openings, closures, funding shocks; event study for pre-trends; individual linkage where available.	No change after provision shocks, or failed parallel-trends test.	The infrastructural claim where it is policy-relevant.
P6	C3 vs. exposure model	At matched referral, social-prescribing effect size varies with surrounding cultural vitality, and environment-level measures explain variance that individual dose does not.	Multi-site evaluations with the area index as moderator; pooled individual-participant data.	Effect sizes invariant to provision, and no variance explained beyond individual dose.	The direct contrast. A null favours the exposure model.
P7	C3	Areas scoring higher on the index host measurably more generative coupling.	Area-sampled ecological recording against the pre-registered supply-side index.	No relation between index score and measured coupling.	Convergent validity of the index; P5 and P6 lose their interpretation.

## Data Availability

No new data were created or analysed in this study.

## Abbreviations

The following abbreviations are used in this manuscript:

ASBA	Anxiety, Stress, Brain-friendly Museum Approach
C^2^	Collective/Coupled Creativity (dyadic generativity unit)
DMN	Default Mode Network
ECN	Executive Control Network
EEG	Electroencephalography
ELSA	English Longitudinal Study of Ageing
fNIRS	Functional Near-Infrared Spectroscopy
RCT	Randomised Controlled Trial
SHARE	Survey of Health, Ageing and Retirement in Europe

## References

[1] Zeki S. (1999). Inner Vision: An Exploration of Art and the Brain.

[2] Chatterjee A., Vartanian O. (2014). Neuroaesthetics. Trends Cogn. Sci..

[3] Salimpoor V.N., Benovoy M., Larcher K., Dagher A., Zatorre R.J. (2011). Anatomically distinct dopamine release during anticipation and experience of peak emotion to music. Nat. Neurosci..

[4] Vessel E.A., Starr G.G., Rubin N. (2012). The brain on art: Intense aesthetic experience activates the default mode network. Front. Hum. Neurosci..

[5] Vanutelli M.E., Banzi A., Cicirello M., Folgieri R., Lucchiari C. (2025). Predicting State Anxiety Level Change Using EEG Parameters: A Pilot Study in Two Museum Settings. Brain Sci..

[6] Banzi A., Sacco P.L., Vanutelli M.E., Lucchiari C. (2026). The Museum as a Mindful Space: Reducing Visitors’ Stress and Anxiety Levels Through the ASBA Protocol. Behav. Sci..

[7] Mastandrea S., Maricchiolo F., Carrus G., Giovannelli I., Giuliani V., Berardi D. (2019). Visits to figurative art museums may lower blood pressure and stress. Arts Health.

[8] Grossi E., Tavano Blessi G., Sacco P.L. (2019). Magic moments: Determinants of stress relief and subjective wellbeing from visiting a cultural heritage site. Cult. Med. Psychiatry.

[9] Dupuy E.G., Vincent T., Lecchino C., Boisvert A., Trepanier L., Nadeau S., de Guise E., Bherer L. (2024). Prefrontal engagement predicts the effect of museum visit on psychological well-being: An fNIRS exploration. Front. Psychiatry.

[10] Stellar J.E., Priewe S., Lidhar N.K., Martin L. (2026). Viewing art as a pathway to psychological well-being and physical health. Appl. Psychol. Health Well-Being.

[11] Fancourt D., Steptoe A. (2019). The art of life and death: 14 year follow-up analyses of associations between arts engagement and mortality in the English Longitudinal Study of Ageing. BMJ.

[12] Fancourt D., Finn S. (2019). What Is the Evidence on the Role of the Arts in Improving Health and Well-Being? A Scoping Review.

[13] Fancourt D., Steptoe A. (2019). Cultural engagement and mental health: Does socio-economic status explain the association?. Soc. Sci. Med..

[14] Løkken B.I., Merom D., Sund E.R., Krokstad S., Rangul V. (2021). Association of engagement in cultural activities with cause-specific mortality determined through an eight-year follow up: The HUNT Study, Norway. PLoS ONE.

[15] Yang L., Zhang N., Zhang Z. (2025). The impact of public cultural participation on public health efficiency: Empirical analysis from Chinese cities. Front. Public Health.

[16] Moss J.L., Hearn M., Cuffee Y.L., Wardecker B.M., Kitt-Lewis E., Pinto C.N. (2023). The role of social cohesion in explaining rural/urban differences in healthcare access and health status among older adults in the mid-Atlantic United States. Prev. Med..

[17] Gao Q., Bone J.K., Bu F., Paul E., Sonke J.K., Fancourt D. (2023). Associations of social, cultural, and community engagement with health care utilization in the US Health and Retirement Study. JAMA Netw. Open.

[18] Van de Cruys S., Wagemans J. (2011). Putting reward in art: A tentative prediction error account of visual art. i-Perception.

[19] Van de Cruys S., Frascaroli J., Friston K. (2024). Order and change in art: Towards an active inference account of aesthetic experience. Philos. Trans. R. Soc. B.

[20] Osiurak F., Federico G., Fournel A., Gaujoux V., Lamberton F., Ibarrola D., Rossetti Y., Lesourd M. (2025). Shaping the physical world to our ends through the left PF technical-cognition area. eLife.

[21] Bluet A., Reynaud E., Federico G., Bryche C., Lesourd M., Fournel A., Lamberton F., Ibarrola D., Rossetti Y., Osiurak F. (2025). The technical-reasoning network is recruited when people observe others make or teach how to make tools: An fMRI study. iScience.

[22] Cosgrove A.L., Diaz M.T., DiStefano P.V., Kenett Y.N., Beaty R.E. (2026). Associative Thinking and Creative Ability in Older Adulthood. Creat. Res. J..

[23] Friston K., Frith C. (2015). A duet for one. Conscious. Cogn..

[24] Luchini S.A., Volle E., Beaty R.E. (2025). The role of the default mode network in creativity. Curr. Opin. Behav. Sci..

[25] Beaty R.E., Benedek M., Silvia P.J., Schacter D.L. (2016). Creative cognition and brain network dynamics. Trends Cogn. Sci..

[26] Christoff K., Irving Z.C., Fox K.C.R., Spreng R.N., Andrews-Hanna J.R. (2016). Mind-wandering as spontaneous thought: A dynamic framework. Nat. Rev. Neurosci..

[27] Lucchiari C., Sala P.M., Vanutelli M.E. (2018). Promoting Creativity Through Transcranial Direct Current Stimulation (tDCS). A Critical Review. Front. Behav. Neurosci..

[28] Lucchiari C., Vanutelli M.E., Folgieri R., Banzi A. (2023). BCI Applications to Creativity: Review and Future Directions from Little C to C^2^. Brain Sci..

[29] Hasson U., Ghazanfar A.A., Galantucci B., Garrod S., Keysers C. (2012). Brain-to-brain coupling: A mechanism for creating and sharing a social world. Trends Cogn. Sci..

[30] Dikker S., Wan L., Davidesco I., Kaggen L., Oostrik M., McClintock J., Rowland J., Michalareas G., Van Bavel J.J., Ding M. (2017). Brain-to-Brain Synchrony Tracks Real-World Dynamic Group Interactions in the Classroom. Curr. Biol..

[31] Lucchiari C., Sala P.M., Vanutelli M.E. (2019). The effects of a cognitive pathway to promote class creative thinking. An experimental study on Italian primary school students. Think. Ski. Creat..

[32] Reche I., Perfectti F. (2020). Promoting Individual and Collective Creativity in Science Students. Trends Ecol. Evol..

[33] Laroche J., Bachrach A., Noy L. (2024). De-sync: Disruption of synchronization as a key factor in individual and collective creative processes. BMC Neurosci..

[34] Acar O.A., Tuncdogan A., van Knippenberg D., Lakhani K.R. (2024). Collective creativity and innovation: An interdisciplinary review, integration, and research agenda. J. Manag..

[35] Woolley A.W., Chabris C.F., Pentland A., Hashmi N., Malone T.W. (2010). Evidence for a collective intelligence factor in the performance of human groups. Science.

[36] Varela F.J., Thompson E., Rosch E. (1991). The Embodied Mind: Cognitive Science and Human Experience.

[37] Thompson E. (2007). Mind in Life: Biology, Phenomenology, and the Sciences of Mind.

[38] Clark A., Chalmers D. (1998). The Extended Mind. Analysis.

[39] De Jaegher H., Di Paolo E. (2007). Participatory sense-making: An enactive approach to social cognition. Phenomenol. Cogn. Sci..

[40] Kiely B., Croke A., O’Shea M., Geaney D., Shortall C., Maher M., Keogh A., McCombe G., Cullen W. (2022). Effect of social prescribing link workers on health outcomes and costs for adults in primary care and community settings: A systematic review. BMJ Open.

[41] Radford-Smith D.E., Anthony D.C. (2026). From fragmentation to integration: Can social prescribing bridge the gap between multimorbidity and mental health in an ageing population?. BMJ Public Health.

[42] Fancourt D., Steptoe A. (2025). Can social prescribing reach patients most in need? Patterns of (in)equalities in referrals in a representative cohort of older adults in England. Perspect. Public Health.

[43] Klinenberg E. (2018). Palaces for the People: How Social Infrastructure Can Help Fight Inequality, Polarization, and the Decline of Civic Life.

[44] Lucchiari C., Vanutelli M.E., Ferrara V., Folgieri R., Banzi A. (2024). Promoting well-being in the museum: The ASBA project research protocol. Int. J. Health Wellness Soc..

[45] Shaikh M., Tymoszuk U., Williamon A., Miraldo M. (2021). Socio-economic inequalities in arts engagement and depression among older adults in the United Kingdom: Evidence from the English Longitudinal Study of Ageing. Public Health.

[46] Fancourt D., Steptoe A. (2024). The contribution of adult experiences, multimorbidity and positive psychological well-being to social inequalities in health. Oxf. Open Econ..

